# Human Primary Adipocytes Exhibit Immune Cell Function: Adipocytes Prime Inflammation Independent of Macrophages

**DOI:** 10.1371/journal.pone.0017154

**Published:** 2011-03-23

**Authors:** Kees Meijer, Marcel de Vries, Saad Al-Lahham, Marcel Bruinenberg, Desirée Weening, Martijn Dijkstra, Niels Kloosterhuis, Roelof Jan van der Leij, Han van der Want, Bart-Jan Kroesen, Roel Vonk, Farhad Rezaee

**Affiliations:** 1 Center for Medical Biomics, University Medical Center Groningen, University of Groningen, Groningen, The Netherlands; 2 Department of Medical Genetics, University Medical Center Groningen, University of Groningen, Groningen, The Netherlands; 3 Department of Pathology and Medical Biology, University Medical Center Groningen, University of Groningen, Groningen, The Netherlands; 4 Cell Biology, Center for Imaging, University Medical Center Groningen, University of Groningen, Groningen, The Netherlands; Mayo Clinic College of Medicine, United States of America

## Abstract

**Background:**

Obesity promotes inflammation in adipose tissue (AT) and this is implicated in pathophysiological complications such as insulin resistance, type 2 diabetes and cardiovascular disease. Although based on the classical hypothesis, necrotic AT adipocytes (ATA) in obese state activate AT macrophages (ATM) that then lead to a sustained chronic inflammation in AT, the link between human adipocytes and the source of inflammation in AT has not been in-depth and systematically studied. So we decided as a new hypothesis to investigate human primary adipocytes alone to see whether they are able to prime inflammation in AT.

**Methods and Results:**

Using mRNA expression, human preadipocytes and adipocytes express the cytokines/chemokines and their receptors, MHC II molecule genes and 14 acute phase reactants including C-reactive protein. Using multiplex ELISA revealed the expression of 50 cytokine/chemokine proteins by human adipocytes. Upon lipopolysaccharide stimulation**,** most of these adipocyte-associated cytokines/chemokines and immune cell modulating receptors were up-regulated and a few down-regulated such as (ICAM-1, VCAM-1, MCP-1, IP-10, IL-6, IL-8, TNF-α and TNF-β highly up-regulated and IL-2, IL-7, IL-10, IL-13 and VEGF down-regulated. In migration assay, human adipocyte-derived chemokines attracted significantly more CD4+ T cells than controls and the number of migrated CD4+ cells was doubled after treating the adipocytes with LPS. Neutralizing MCP-1 effect produced by adipocytes reduced CD4+ migration by approximately 30%.

**Conclusion:**

Human adipocytes express many cytokines/chemokines that are biologically functional. They are able to induce inflammation and activate CD4+ cells independent of macrophages. This suggests that the primary event in the sequence leading to chronic inflammation in AT is metabolic dysfunction in adipocytes, followed by production of immunological mediators by these adipocytes, which is then exacerbated by activated ATM, activation and recruitment of immune cells. This study provides novel knowledge about the prime of inflammation in human obese adipose tissue, opening a new avenue of investigations towards obesity-associated type 2 diabetes.

## Introduction

An imbalance or dysfunction of adipose tissue (AT) contributes to obesity-induced chronic inflammation, which in turn results in energy metabolism disorders such as insulin resistance (IR), type 2 diabetes, inflammation and cardiovascular disease [1]–[11]. Although the involvement of adipocytes in energy regulation is clear, little is known about their role in the occurrence of inflammation in AT, which is assumed to be important in the development of type 2 diabetes. Notwithstanding the fact that AT is recognized as an immune organ and adipose tissue macrophages (ATM) appeared to be the major source of inflammation in AT [10]–[15], there has been no systematic study on the role of adipocytes in inflammation. We hypothesized that obesity led to adipocyte dysfunction, which in turn primes an inflammation state, although this priming is not due to adipose tissue-infiltrated macrophages. In contrast, inflamed AT adipocytes (ATA) may promote the infiltration of ATM and change the macrophages with a resident character (anti-inflammatory macrophages called M2 macrophages) into migrated macrophages (pro-inflammatory macrophages called M1 macrophages) [11] through a paracrine action, thereby establishing a vicious environment that exacerbates the inflammatory pattern in the AT and in particular, in adipocytes. It is also possible that inflamed AT adipocytes (ATA) may provide the signal(s) that lead to the generation of the migrated macrophages.

To test our hypothesis, we investigated whether the adipocytes synthesize immune-associated components and if so, whether these adipocyte-expressed immune genes and proteins are biologically active and functional. In this study, we used state-of-the-art proteomic, genomic and microscopy techniques.

## Results and Discussion

### Differentiation of pre-adipocytes into adipocytes

**Figure 1:**
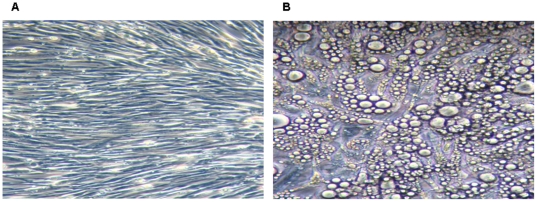
Differentiation of human pre-adipocytes into adipocytes. Panel **A** shows human pre-adipocytes (long, thin and flatted cells without detectable fat organelles). Panel **B** shows that differentiated fat cells (adipocytes) are spherical with differences in fat organelle size. Most space in adipocytes is occupied by fat organelles.

**Figure 2:**
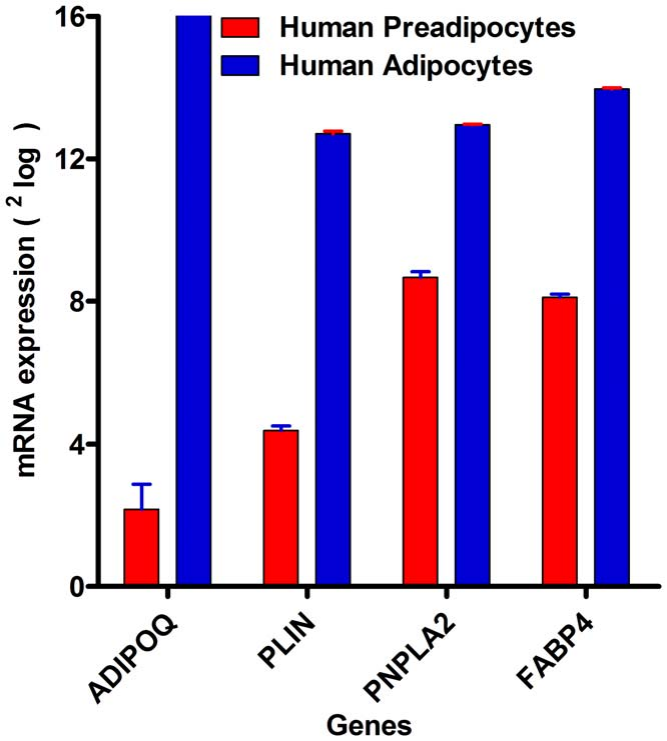
The differentiation markers for adipocytes. Illumina beadarray was used to screen adipocytes differentiation markers. These markers are Adiponectin (*AdipoQ*), perilipin (*PLIN*), adipose triglyceride lipase (*PNPLA2*) and fatty acid binding protein (*FABP4*). Gene expression was expressed as ^2^log (e.g. the difference between pre-adipocytes and adipocytes for adiponectin expression is approximately 10, thus the true difference is 2^10^ ( = 1024-fold) and this is shown on the y-axis).

**Figure 3:**
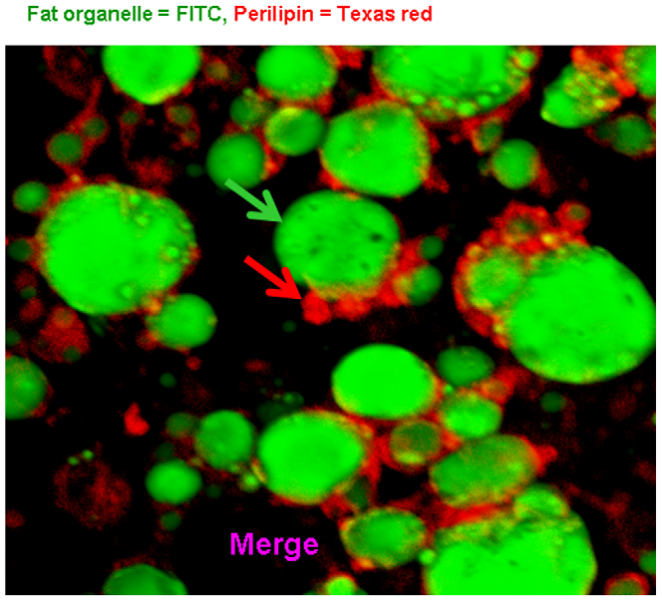
Confocal laser scan microscopy (CLSM) analysis of Perilipin in adipocytes. CLSM was used to validate and localize perilipin protein in human adipocytes as shown in figure 3. Fat organelles ( =  lipid droplets) were stained with FITC (green color), indicated by green arrow, and Perilipin was visualized with Texas red (red color) shown by red arrow. Perilipin was localized at the surface of fat organelles.

To obtain a homogenous adipocyte fraction, human primary pre-adipocytes were differentiated into adipocytes (adipogenesis), which was morphologically monitored (**Figure 1 A–B**). The efficiency of differentiation reached approximately 90–95% as assessed by morphology, indicating that the adipocytes could be considered as a homogenous fraction. In addition, Illumina BeadArray showed a significant up-regulation of 4 mRNA markers of adipocytes, i.e. adiponectin (*ADIPOQ; 1000-fold*, P<0.0008), perilipin (*PLIN; 500-fold*, P<0.000006), adipose triglyceride lipase (*PNPLA2; 32-fold*, P<0.000009), and fatty acid binding protein 4 (*FABP4; 128-fold*, P<0.000006). All these four genes are involved in lipid metabolism and the hydrolization of triglycerides [4], [5], [16], [17] and were highly expressed in adipocytes compared to pre-adipocytes (**Figure 2**). Perilipin (the hallmark of fat organelle  =  droplet fat), was also shown and confirmed at protein level by immunofluorescent confocal microscopy (CLSM) (**Figure 3**).

### Illumina (mRNA) BeadArray

**Figure 4:**
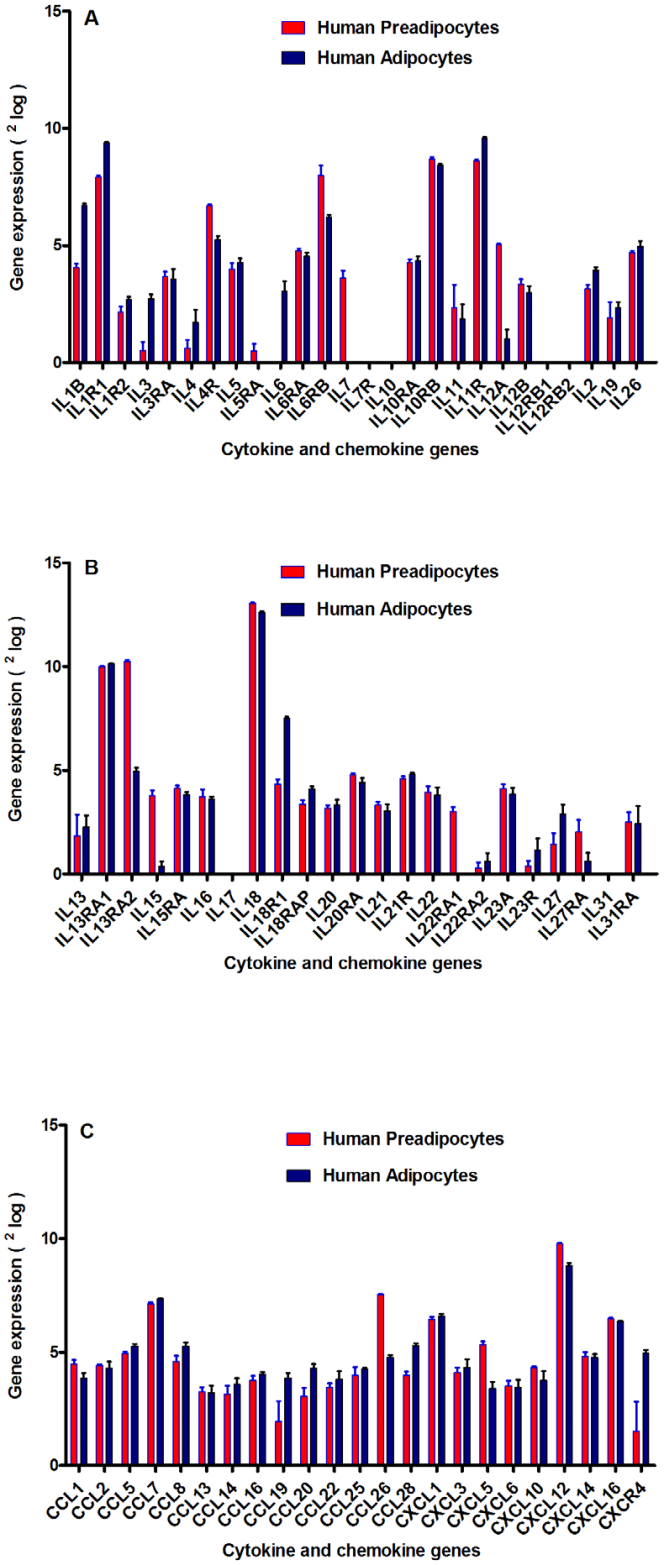
The expression of immune-associated components (cytokine and chemokine genes) by human primary adipocytes. Figure 4 A–C shows cytokines and chemokines and their receptor gene expression by human adipocytes, using Illumine BeadArray. Illumina gene profiling analysis shows the expression of cytokines and chemokines in human pre- adipocytes (**red bars**) and adipocytes (**blue bars**). Gene expression was expressed as ^2^log and is shown on the y-axis. Illumina results were derived from two independent experiments. Each experiment was done in duplicate.

**Figure 5:**
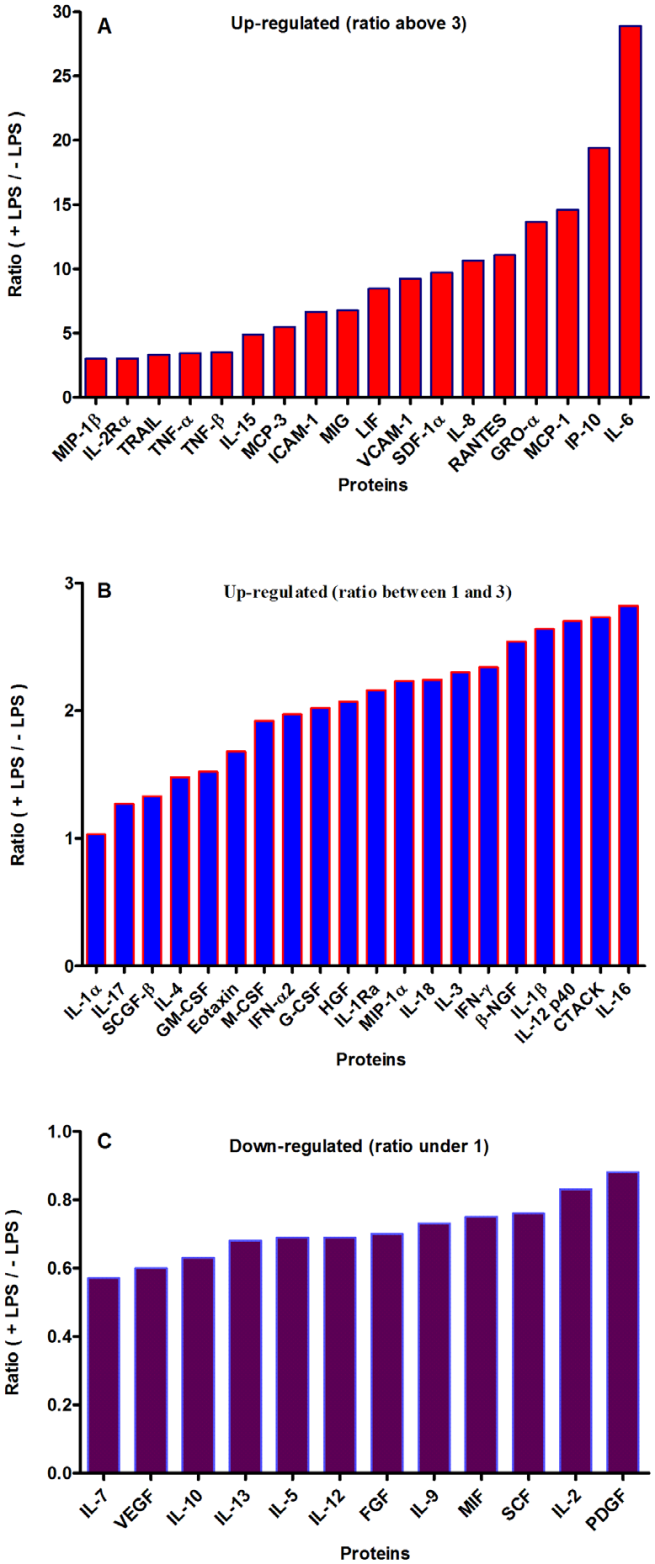
A comparison analysis of 50 cytokines/chemokines between adipocytes and adipocytes treated with LPS. Figures 5 A and B show up-regulation above ratio 3 (**panel A**) and from ratio 1 to 3 (**panel B**) and down-regulation (**panel C**, ratio under 1), respectively, of cytokine and chemokine proteins before and after lipopolysaccharide (LPS) stimulation (200 ng/ml with a final concentration of 1 µg for 24 h), using a multiplex ELISA cytokine/chemokine protein BeadArray. Protein expression was expressed as the ratio of LPS-treated/untreated and is shown on the y-axis. Preadipocytes are shown in red bars and adipocytes in blue bars.

**Figure 6:**
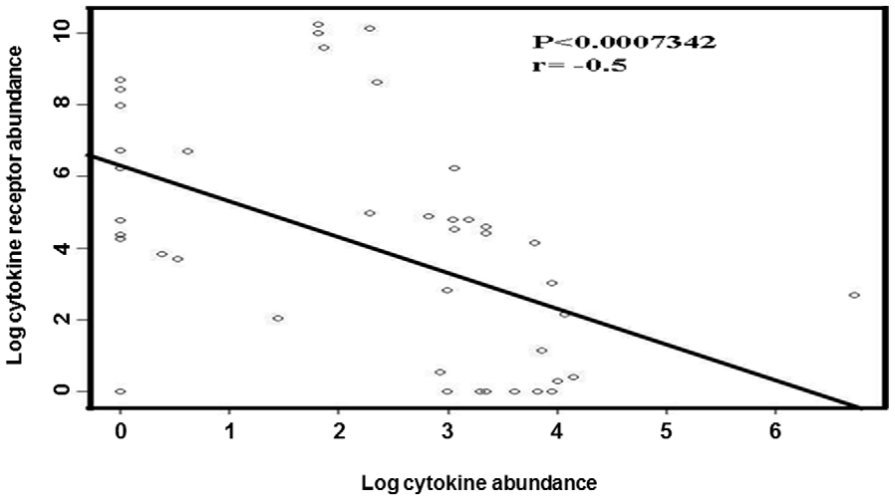
The correlation between cytokines/chemokines and their receptors. Figure 6 shows a significant negative correlation between cytokines/chemokines and their receptor genes. Illumina results were derived from two independent experiments. Each experiment was done in duplicate.

After establishing our experimental set up, we determined immune-expressed genes in pre-adipocytes and adipocytes by Illumina BeadArray. Intriguingly, both pre-adipocytes and adipocytes expressed the majority of known immune-associated cytokine and chemokine genes (**Figure 4 A–C**), either pro-inflammatory like *TNF-α, IL-17, IL-19, CCL19* and *CCL22* [6], [9], [11], [12], [13], [15] or anti-inflammatory such as *IL-13* [18], [19]. Based on the classical hypothesis, ATM is activated by triggers like lipopolysaccharide (LPS), which in turn produce pro-inflammatory chemokines and cytokines such as TNF-α, IL-6 and monocyte chemoattractant protein-1 (MCP-1; M1 macrophages). M1 macrophages could lead to lower insulin sensitivity in tissue targets. However, alternative activated macrophages (M2 macrophages) appeared to be occurring via the production of the anti-inflammatory IL-13 (**Figure 5 C**) and IL-4 cytokine proteins by the adipocytes in a homeostatic state. Moreover, it has been speculated that these ATM2 are able to increase insulin sensitivity via the production of anti-inflammatory IL-10 protein [5], [11], [18], (**Figure 5 C**). Although no large differences were observed between pre-adipocytes and adipocytes with respect to expression of immune genes, *IL-6* (pro-inflammatory) was only expressed in adipocytes, whereas *IL-7* (crucial for T-cell homeostasis and could be considered as anti-inflammatory) was only expressed in pre-adipocytes (**Figure 4 A**). Since human primary pre-adipocytes and adipocytes expressed substantial amounts of mRNA of many cytokines and chemokines, these immune genes seem to be an intrinsic property of this cell type. In the mRNA BeadArray we also found that both pre-adipocytes and adipocytes synthesize many cytokine and/or chemokine receptors (**Figure 4 A–C**). Furthermore, a significant negative correlation was seen between cytokine expression levels and the expression of their corresponding receptors (P<0.0007; r = −0.5), except for IL-18, for which both the cytokine and its receptor mRNA were highly expressed (**Figure 6**). This suggests that the efficiency of ligand-receptor interaction depends on the concentration.

### Proteomics, multiplex ELISA, confocal and immunoelectron microscopy

**Figure 7:**
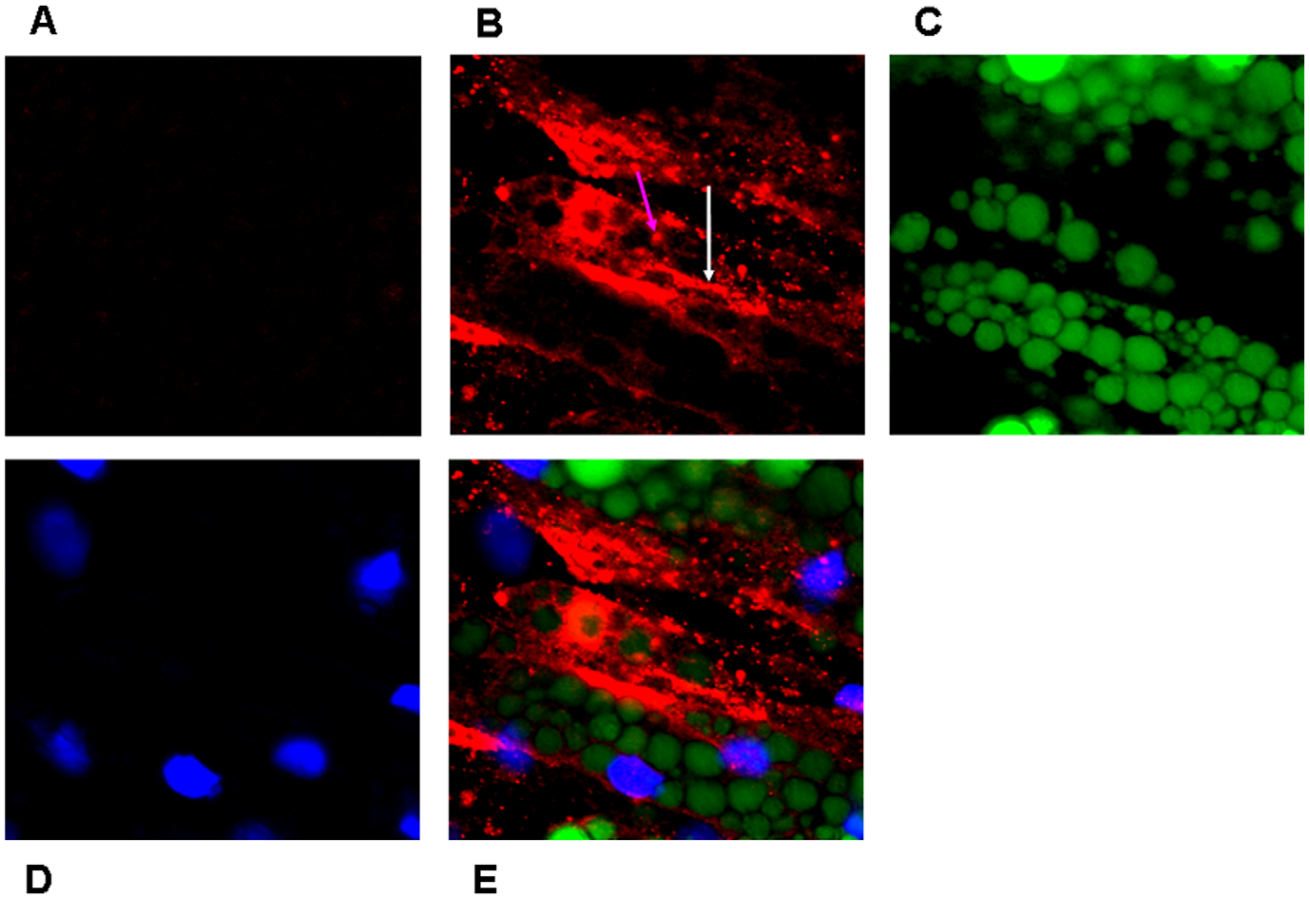
The localization and visualization of C-RP in the human adipocytes using confocal microscopy strategy. Confocal microscopy analysis was used to localize C-reactive protein (CRP) in the human adipocytes. An adipocyte wet slide was stained only with IgG isotype and alexa 647 coupled goat anti-mouse **(panel A)**; considered as a negative control). This slide was not incubated with primary monoclonal antibody (Mab) against human CRP. Another adipocyte wet slide was also stained with Mab against human CRP and bound antibodies were displayed with alexa 647 coupled goat anti-mouse (panel **A and E**; red color). Fat organelle (lipid droplets) was visualized with FITC (**C**; green color), and adipocytes were stained with DAPI to detect nuclei (panel **D**; blue color). **Panel E** shows a merging of all three labels. Fluorescent labeling was used for all detections. CRP was found to be positive in cytoplasm and plasma membrane of human adipocytes and this confirmed the gene expression of CRP in both pre-adipocytes and adipocytes obtained by Illumina BeadArray (**Table 1**). White and red arrows indicate the presence of CRP on cytoplasm and plasma membrane, respectively (**panel B**).

**Figure 8:**
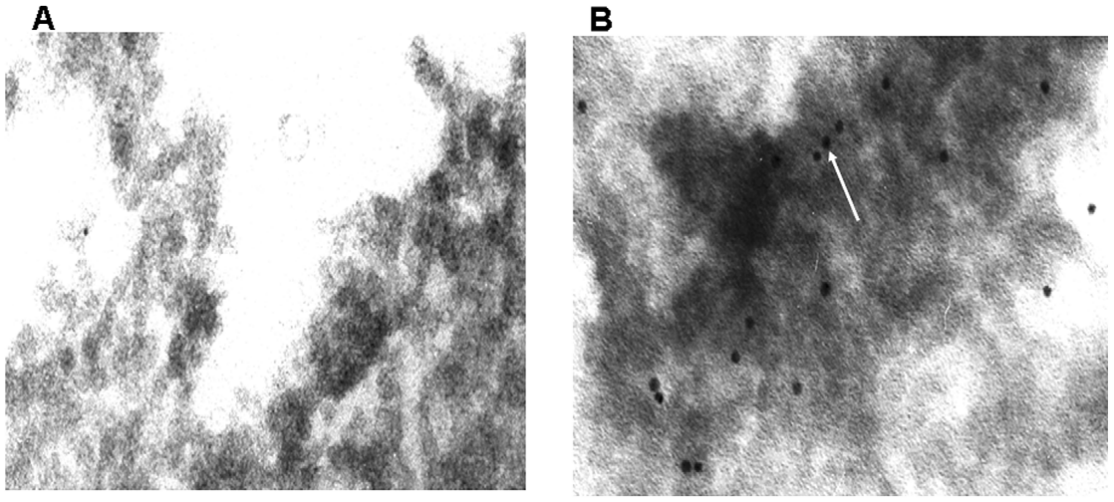
Localization of C-RP in human adipocytes using Immunoelectron microscopy. Panel **A** served as a negative control and this slide was not incubated with primary monoclonal antibody (Mab) against human CRP, but with IgG isotype. **Panel B** slide was labeled with Mab against human CRP and bound antibodies were displayed with coupled goat anti-mouse 15 nm gold particles. White arrow shows one CRP gold-stained particle (Black dot).

A proteomics approach was used to further validate the gene expression data. So far, all proteomics studies aiming at analyzing the adipocyte proteome have been performed in murine 3T3-L1 cells or in rat adipose cells [20]. A proteomic study of human primary adipocytes has not been reported before. Hence, a combination of different proteomics techniques, including LTQ-Orbitrap XT and Q-STAR, were used to analyze the production of cytokines and chemokines by human adipocytes. Although this approach led to the identification of approximately 1800 proteins, no cytokine or chemokine proteins were identified except for acute phase proteins. This is presumably due to their low abundancy and the current mass spectrometry analyses/sample processing is apparently not sensitive enough to detect such low abundancies. Therefore, a very sensitive, multiplex ELISA with a panel of 50 cytokine and chemokine proteins was used to analyze whether these proteins are synthesized by human adipocytes. This resulted in the detection of all 50 cytokines and chemokines present in this panel in human adipocytes (**Figure 5** **A–C**). To investigate whether these cytokines and chemokines are biologically active, the differentiated adipocytes were stimulated with LPS (200 ng/ml with a final concentration of 1 µg for 24 h). Adipocytes treated with LPS led to the up-regulation (Figure 5 A and B) of the majority of cytokines and chemokines, while a small fraction was down-regulated (**Figure 5** **C**). All of the up-regulated cytokine and chemokine proteins were pro-inflammatory (e.g. IL-6, TNF-α, MCP-1, IL-8) (Figure 5 A), whereas a few down-regulated cytokines and chemokines were established anti-inflammatory (e.g. IL-10 and IL-13) (Figure 5 C). These results indicate that adipocyte-produced cytokines and chemokines are biologically active and might have a physiological relevance. Although no adipocyte-associated cytokines and chemokines were detected by the proteomics approach, this approach resulted in the identification of 11 known acute phase proteins (**Table 1**). Illumina BeadArray of human adipocytes confirmed the expression of the majority of these acute phase proteins at gene level (**Table 1**). A combined analysis of proteomics and Illumina BeadArray led to the identification of 14 established [21**]**-[****24] acute phase proteins (**Table 1**). Intriguingly, this approach also revealed the presence of C-reactive protein (CRP) (**Table 1**), which is considered to be the most significant marker of inflammation. Both pre-adipocytes and adipocytes expressed the *CRP* gene, which was confirmed by RT-PCR analysis (data not shown). The fact that pre-adipocytes synthesize CRP suggests that this gene was an intrinsic component of this cell. In addition, immunofluorescent confocal microscopy analysis using CRP-specific monoclonal antibodies [21] confirmed a substantial production of CRP at the protein level in adipocytes (**Figure 7** **A–E**). Immunofluorescent images were confirmed with immunoelectron microscopy using the same monoclonal antibody (**Figure 8** **A–B**). We focused our attention on C-RP because it plays an important role in host defense (immune-associated function) and elevated C-RP levels are positively correlated to type 2 diabetes. CRP is also considered to be a prognostic marker in many diseases [22]–[24].

**Table 1:** The expression of acute phase genes and proteins by human primary adipocytes.

Protein name	Accession No.	Gene No.	Gene name	Secretion	PI	Mw (Da)
Alpha-2-HS-glycoprotein	P02765	112910	AHSG	Yes	5.43	39325
Albumin	P02768	113576	ALB	Yes	5.92	69367
Transferrin	P02787	136191	TF	Yes	6.81	77050
Fibronectin	P02751	2506872	FN1	Yes	5.45	282607
Plasminogen activator inhibitor-1	P05121	129576	Serpin A1	Yes	6.68	45060
Serum amyloid A-4	P35542	548885	SAA-4	Yes	9.27	14807
Complement C3	P01024	120000000	C3	Yes	6.21	87148
Ferritin heavy chain	P02794	120516	FTH1	Yes	5.31	21226
Angiotensiogen (SERPINA8)	P01019	113880	AGT	Yes	5.87	53154
Plasma protease C1 inhibitor	P05155	124096	SERPING1	Yes	6.09	55154
Alpha-antichymotrypsin (ACT)	P01011	112874	SERPINA3	Yes	5.33	47651
Interleukin-1 receptor antagonist	P18510	124312	IL1RN	Yes	5.83	20055
Granulocytes-colony stimulating factor	P09919	4503079	CSF3	Yes	5.61	22293
C-Reactive protein	P02741	56000000	C-RP	Yes	5.45	35039

Table 1 represents the 14 acute phase proteins and genes expressed by adipocytes, using Illumina BeadArray, protein array (Q-STAR and LTQ-Orbitrap XL) and confocal microscopy.

### Cell migration of CD4+ T cells, expression of MHC class II, and co-stimulation of genes by human adipocytes

**Figure 9:**
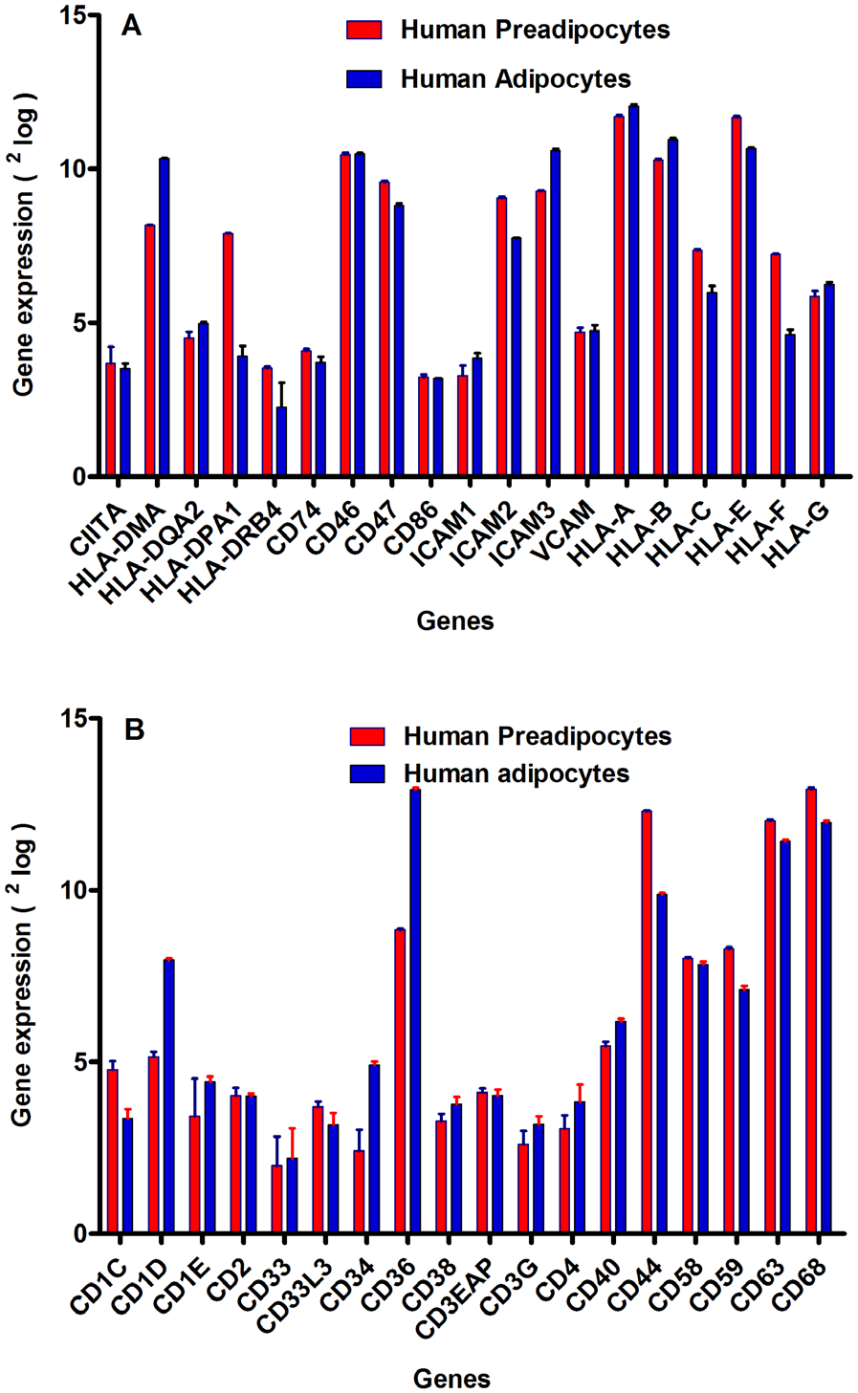
The expression of the MHC class II genes, receptors involved in (co)stimulation of T cells and immune-associated cluster differentiation (CD) by human primary pre-adipocytes and adipocytes. **Panel A** shows the expression of MHC class II genes and some genes involved in co-stimulation of T cells in human pre-adipocytes (**red bars**) and adipocytes (**blue bars**). Gene expression was given as ^2^log and shown on the y-axis. Illumina results were obtained from *two independent* experiments. Each experiment was done in *duplicate*, thus, each sample was measured four times. **Panel B** shows CD gene expression by human adipocytes, using Illumina BeadArray. Illumina gene profiling analysis shows the expression of CD genes in human pre-adipocytes (**red bars**) and adipocytes (**blue bars**). Gene expression was expressed as ^2^log and shown on the y-axis.

**Figure 10:**
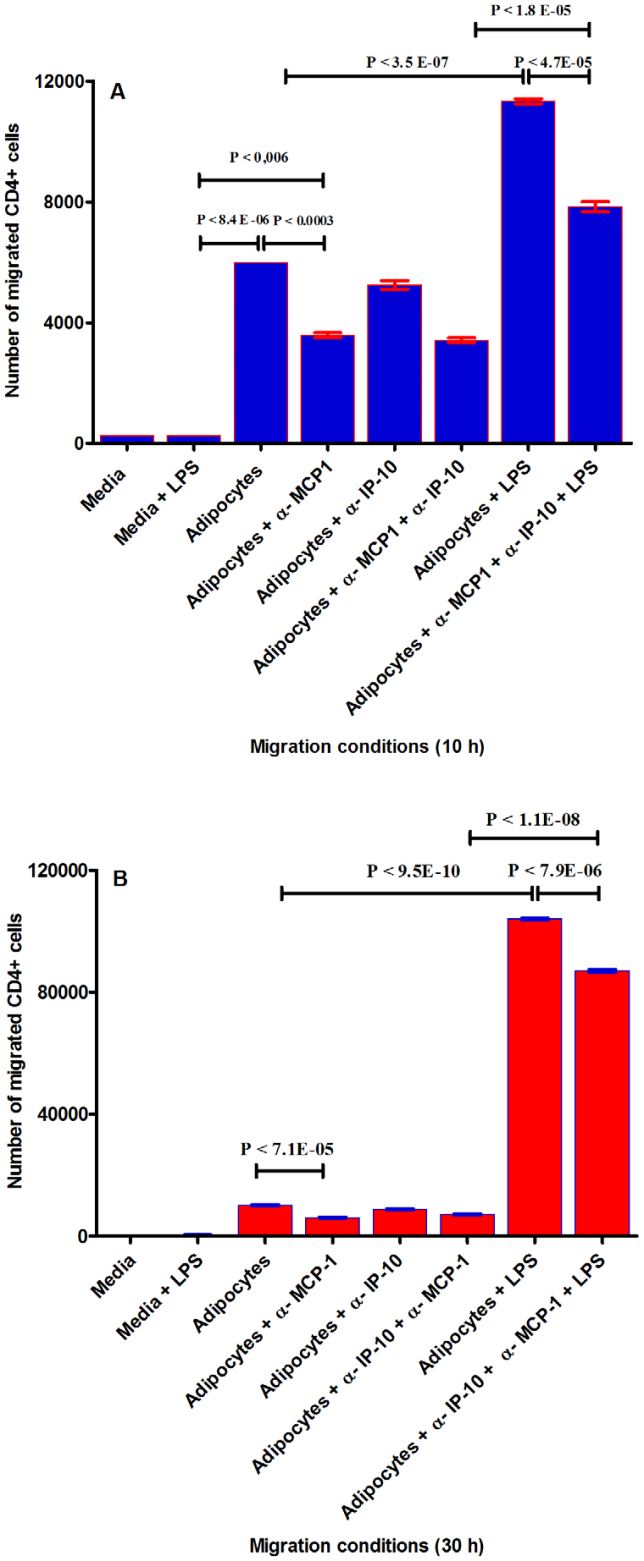
The activation of CD4+ cells by human adipocytes. **Panel A** shows the analysis of CD4+ cells migration caused by adipocytes-produced chemokines after 10 h incubation. The lower compartment (insert) contained attached adipocytes treated with LPS (200 ng/ml) or untreated, as well as antibodies against MCP-1 and IP-10 (2 µg/ml) to neutralize the effect of these two chemo-attractants. The upper chamber (insert) contained purified and rested CD4+ T cells. The cells were incubated at 37°C for 10 h. The inserts were then removed and the cells migrated toward the adipocytes were collected in culture medium without serum. The number of migrated cells was obtained by cell counting using a Bürker-Türk chamber. This figure represents two independent experiments with similar results (only differ in the number of migrated cells). Each experiment was done in triplicate. Error bars show standard deviations of means for triplicate cultures per condition. The p-value obtained from an independent two-tailed test samples (T-test). P<0.05 was accepted as statistically significant. The controls in the migration assay were 1- culture medium without serum, 2- culture medium serum free with LPS, and culture medium serum free with IgG isotype (this is not shown in **Figure 10** **A** and **10 B**, because this was executed separately and the obtained results were the same as culture medium serum free with LPS). **Panel B** shows the analysis of CD4+ cells migration caused by adipocyte-produced chemokines after 30 h incubation. All cell migration conditions were similar to the conditions described in **panel A**, except that the cells were incubated at 37°C for 30 h.

Together, these results suggest that human primary pre-adipocytes and adipocytes exhibit immune cell-like behavior. In addition, adipocytes were found to express multiple receptors involved in T-cell (co)stimulation, especially upon treatment with LPS. These receptors included adhesion molecules, e.g. vascular cell adhesion molecule 1 (VCAM-1), intercellular adhesion molecule 1–3 (ICAM-1-3), the ligand CD62L (CD34), CD36, CD44, CD47 and CD58; receptors involved in antigen presentation (MHC class II), e.g. C2TA ( =  CIITA), HLADM, HLADP, HLADQ and HLADR, CD1C-D, CD74 and CD4; receptors involved in T-cell co stimulation, e.g. B7.2 (CD86), CD2, CD38, CD40; as well as a significant number of cytokines and chemokines, e.g. MIP-1a, IFN-g, TRAIL, TNF-a, IL-8, RANTES, MCP-1, IP-10 and IL-6 (**Figure 5** **A and B**) and (**Figure 9** **A–B**). It must be noted that many of these proteins have key functions in many aspects of the activation process leading to cell-mediated immunity and, as such, are reminiscent of professional antigen-presenting cells (MHC II molecule) [25]. This observation led us to investigate whether human adipocytes exhibit true immune-cell function. We studied the role of human adipocytes in T-cell migration, which is an important requirement for allowing cell-mediated immunity. Since CD4^+^ T-cells are central to the regulation of cell-mediated immunity [25], [26], we focused specifically on this cell population. It was also noted that pretreatment with LPS significantly up-regulated cytokine and chemokine expression by the adipocytes. We therefore investigated the migration of CD4+ T cells towards human adipocytes (either treated with LPS or untreated) via the cell migration assay in a Boyden chamber [27] with a 5- µm-pore polyethylene terephthalate (PET) membrane (Millipore PET membrane insert for 12-wells,). CD4+ T cells were uploaded in the upper chamber (insert). As shown in **Figure 10** **A**, approximately 6000 CD4+ cells (assessed by counting of CD4+ in a Bürker-Türk chamber) [28] were migrated (after 10 h incubation) from the upper chamber towards the untreated human adipocytes (lower chamber), indicating that adipocyte-associated chemokines are functional. If human adipocytes were treated with LPS, the number of migrated CD4+ T cells approximately doubled (Figure 10 A). We then investigated the role of the two most up-regulated chemokines (MCP-1 and IP-10) upon LPS stimulation (**Figure 5** **A**) in the migration of CD4+ T cells towards adipocytes. The effect of monocyte chemoattractant protein-1 (MCP-1) and IP-10 were neutralized by adding of monoclonal anti-body against human MCP-1 and IP-10 (α-MCP and α-IP-10) to the adipocytes treated and untreated with LPS. When α-MCP-1 was added to the adipocytes treated with and without LPS, the migration of CD4+ T cells dropped significantly by approximately 30%. Although the neutralization of IP-10 exhibited a slight reduction of CD4+ migration, this decrease was significant for this chemokine. In addition to two independent migration experiments with similar results, we also performed one migration assay with similar conditions but with an incubation time of 30 h. Intriguingly, we found that adipocytes treated with LPS increase CD4+ cell migration by approximately ten-fold in a time-dependent manner (**Figure 10** **B**). This could be due to a potentially increased concentration of adipocyte-associated chemokines after 30 h incubation with LPS.

### Conclusion

We have revealed that adipocytes express cytokines, chemokines, multiple receptors, cell adhesion molecules and MHC class II family involved in (co)stimulation of T cell activation. Since adipocyte-associated immune components (genes and proteins) responded to LPS stimulation, these immune components are biologically active with a physiological relevance in adipocytes. The functionality of these adipocyte-synthesized components was confirmed by assessing the role of adipocytes in immune cell migration, using CD4+ T-cells as responder cells. Cell migration assay showed that adipocytes are able to activate CD4+ T cells. Thus, adipocytes dysfunction promotes inflammation via its own cytokine and chemokine synthesis machinery and is not dependent on invading macrophages. This immune cell function of adipocytes could play a direct role in the etiology of insulin resistance, type 2 diabetes and cardiovascular disease.

## Materials and Methods

### Cell culture

All human primary adipocytes were purchased from Lonza and PromoCell cell culture companies.

Human primary preadipocytes (Lonza) were grown to confluence in Preadipocyte growth medium provided by PromoCell in 5% CO2 at 37°C. When the cells reached confluence, the medium was replaced with preadipocyte differentiation FCS free medium supplemented with a cocktail (provided by promoCell) of 0.5 mM 3-isobutyl-1-methyxanthine, 400 ng/ml dexamethasone, 0.5 ug/ml bovine insulin, L-thyroxin 9 ng/ml, ciglitazone 3 ug/ml and d-biotin 8 ug/ml for 72 hours. Three days after the initiation of the cells differentiation, the differentiation media were replaced with adipocyte nutrition medium containing 3% FCS, d-biotin 8 ug/ml, 0.5 ug/ml bovine insulin and 400 ng/ml dexamethasone for a period of 16 days (terminal phase of differentiation). At this point, the adipocyte nutrition medium was withdrawn and the cells were washed 5 times with the differentiation media. After wash steps, the cells were incubated with the differentiation medium for 2 days and medium collected containing secretion proteins of human adipocytes. The collected media were concentrated up to 500 X (8.0 ug/ul), using a cut-off filter of 3 kDa (Vivian-science). This collected media was used for further experiments.

The differentiation medium was also concentrated up to 4000 X, using a cut-off filter of 3 kDa and served as control medium to check medium composition in this study. The study had the approval of the local Medical ethical committee (University Medical University Groningen).

### 1-Dimensional electrophoresis (1-DE) and In-gel digestion

From the collected media containing secreted human adipocytes proteins 160 µg protein was separated by SDS-PAGE on pre-cast 4–12% polyacrylamide gradient gels (Invitrogene). Approximately 3×10^7^ cells were lyzed and subsequently proteins were extracted in two different fractions and concentrated. 1.5 mg protein was separated by SDS-PAGE on a big 12% polyacrylamide gel (20 cm × 20 cm). Proteins were reduced with dithiothreitol (DTT), and prepared for electrophoresis according to the manufacturer's instructions (GE Healthcare). Protein bands were visualized using commassie blue staining kit (Invitrogen). The whole lane was divided into 16 slices (in case of pre-cast 4–12% polyacrylamide gradient gels) and 40 slices (in case of big size gels). The 16 and 40 gel pieces were distained, washed and after treatment with DTT and iodoacetamide alkylation, Protein-containing gel slices were treated with trypsin (modified sequencing grade; Promega) and extracted according to the manufacturer's instructions (Promega).

### Isoelectric focusing of peptides and In-solution digestion

Media with adipocyte secreted proteins (100 µl; equivalent to 800 µg of protein) were digested with 8 µg modified trypsin for overnight at 37°C. After tryptic digestion, total volume of sample was adjusted into 700 µl by adding IEF compatible solutions. The non-linear immobilized pH gradient strips (GE Healthcare, pH 3–11) were rehydrated with sample for overnight. IEF was performed directly after rehydration in an Ettan IPGPhor 3 cell (GE Healthcare). After IEF the strip were cut in 11 slices and peptides were extracted according to the manufacturer's instructions (Promega).

### Q-STAR-XL

1-DE and IEF samples were reconstituted with 40 µl water containing 0.1% TFA. 5–8 uL of this solution was used for analysis. 1-DE nanoLC–ESI–MS–MS analysis was performed on an integrated nanoLC system (Agilent) comprising a binary gradient pump with a cooled auto sampler, an capillary pump for loading and washing the trap column, a column switching module configured for trap plus analytical capillary column, and a Q-Star XL API mass spectrometer (Applied Biosystems, MDS Sciex, Framingham, USA) fitted with nano-LC sprayer and a stainless steel emitter (Proxeon, Odense,DK) and operated under Analyst QS 1.1 control. Injected samples were first trapped and desalted isocratically on an LC-Packings PepMap C18 µ-Precolumn Cartridge (5 µm, 300 µm I.D. ×1 mm; Dionex, Sunnyvale, CA, USA) for 5 min with 0.1% formic acid delivered by the auxiliary pump at 20 µl/min after which the peptides were eluted from the trap column and separated on an analytical C18 capillary column (5 cm × 75 µm, C18 Pepmap Dionex) connected in-line to the mass spectrometer, at 300 nl/min using a 93 min gradient of 5–50% acetonitrile in 0.1% formic acid. The Q-Star XL mass spectrometer was operated in information-dependent acquisition (IDA) mode. In MS mode, ions were screened from *m*/*z* 300 to 1100, and MS–MS spectra were acquired from *m*/*z* 65 to 2000 in standard acquisition mode, each acquisition cycle was comprised of a 1s MS and 3 2s MS–MS scans. In IDA mode the three most intense peaks (with charge 2+, 3+) were selected and MS–MS spectra acquired when their intensities exceeded 30 counts and after 2 acquisitions dynamically excluded for 45 seconds with 50 millimass units (mmu) tolerance.

### LTQ Orbitrap XL

Samples were analyzed by nanoLC–MS/MS on an Ultimate 3000 system (Dionex, Amsterdam, The Netherlands) interfaced on-line with a LTQ–Orbitrap-XL mass spectrometer (ThermoFisher Scientific, San Jose, CA). Re-dissolved peptides were loaded onto a 5 mm × 300 µm i.d. trapping micro column packed with C18 PepMAP100 5 µm particles (Dionex) in 0.1% FA at the flow rate of 20 µL/min. Upon loading and washing, peptides were back-flush eluted onto a 15 cm × 75 µm i.d. nanocolumn, packed with C18 PepMAP100 3 µm particles (Dionex). The following mobile phase gradient was delivered at the flow rate of 300 nL/min: 5–50% of solvent B in 93 min;; 50–80% B in 5 min; 80% B during 10 min, and back to 5% B in 5 min. Solvent A was 100∶0 H_2_O/acetonitrile (v/v) with 0.1% formic acid and solvent B was 10∶90 H_2_O/acetonitrile (v/v) with 0.1% formic acid. Peptides were infused into the mass spectrometer via dynamic nanospray probe (ThermoElectron Corp.) with a stainless steel emitter (Proxeon, Odense, DK). Typical spray voltage was 1.6 kV with no sheath and auxiliary gas flow; ion transfer tube temperature was 200°C. Mass spectrometer was operated in data-dependent mode. The automated gain control (AGC) was set to 5×10^5^ charges and 1×10^4^ charges for MS/MS at the linear ion trap analyzer. DDA cycle consisted of the survey scan within *m*/*z* 300–1600 at the Orbitrap analyzer with target mass resolution of 60,000 (FWHM, full width at half maximum at m/z 400) followed by MS/MS fragmentation of the five most intense precursor ions under the relative collision energy of 35% in the linear trap. Singly charged ions were excluded from MS/MS experiments, and *m*/*z* of fragmented precursor ions were dynamically excluded for further 90 s. Ion selection threshold for triggering MS/MS experiments set to 500 counts. An activation parameter *q* 0.25 and activation time of 30 ms were applied.

### Data analysis (software)

#### Q-STAR-XL

ProteinPilot software 2.0 (Applied Biosystems) was applied to identify proteins from database searches of mass spectrometry data using uncompressed human database. Methionine oxidation and carboxamidomethylation were chosen as modifications for database search. This software is compatible with spectra generated by Q-STAR.

#### LTQ Orbitrap-XL

Protein identification was performed using the Turbo- SEQUEST algorithm in the BioWorks™ 3.1 software (Thermo Electron) and the uncompressed human database (Swiss Institute of Bioinformatics, Geneva, Switzerland). The following HUPO SEQUEST criteria were selected for high confidence peptide identification: 1- charge state *versus* cross-correlation number (XCorr) and that is XCorr > 1.9 for singly charged ions, XCorr > 2.7 for doubly charged ions, and XCorr > 3.70 for triply charged ions, 2- deltaCn 0.1, 3- peptide probability 0.001, 4-RsP 4- and 5-final score (sf) 0.85.

### Illumina Beadsarray (mRNA array)

All data is MIAME compliant and the raw data has been uploaded to MIAMEXPRESS (Arrayexpress). The website is http://www.ebi.ac.uk/arrayexpress/. The experiment name is: Human primary preadipocytes and adipocytes and ArrayExpress accession number is: E-MEXP–3092.

For Illumina microarray analysis, Total RNA was extracted from two independent human primary adipocytes culture wells (9 cm^2^). The quality and concentration of the RNA were determined by Bioanalyser using the Agilent RNA 6000 Nano kit (Agilent, Amstelveen, The Netherlands).

Illumina TotalPrep RNA Amplification Kit was applied to amplify and label of the RNA. (Applied Biosystems, Nieuwerkerk ad IJssel, The Netherlands). cDNA was made from total RNA and the concentrations were determined in nanodrop. Prepared and analyzed in Illumina laboratories by Illumina personnel. Biotinylated cRNA was prepared using the Illumina RNA Amplification Kit (Ambion, Inc., Austin, TX) according to the manufacturer's directions starting with 200 ng total RNA. Samples were purified using the RNeasy kit (Qiagen, Valencia, CA). Hybridization to the Sentrix HumanRef-8 V2 Expression BeadChip array (Illumina, Inc., San Diego, CA, USA), washing and scanning were performed according to the Illumina BeadStation 500 manual (revision C). One BeadChip with eight arrays were used. Slide was scanned immediately.

First line quality check, background correction and utile normalization of the data was done with Beadstudio Expression module v 3.2.7. Statistics and gene lists were generated using Genespring GX 7.3.1 (Agilent). P-values were corrected for multiple testing using Benjamini and Hochberg False Discovery Rates.

### mRNA Expression Analysis

#### RNA isolation

Qiagen Rneasy lipid mini kit (Qiagen, GmbH) was used to isolate RNA from human primary adipocytes according to the manufacturer recommendations. The RNA was solved in 30 µl Rnase-free water and stored at −80°C. Integrity of RNA and concentration was checked using a Nanodrop spectrophotometer (Isogen, Life-science). For the cDNA synthesis 1 µg RNA was used with an end volume of 20 µl. Reverse transcription was performed with Quantitect Reverse Transcription kit (Qiagen GmbH).

#### Real time PCR analysis (RT-PCR)

The relative expression of 30 genes (table 1) was measured with Relative Real-time PCR. Relative Real-time PCR was performed on the AB Prism 7900HT Sequence detector (Applied Biosystems, UK) with a primer concentration of 900 nM, probe concentration of 250 nM and the input of cDNA was 25 ng. The PCR profile consisted of 10 minutes at 95°C, followed by 40 cycles with heating of 95°C for 15 seconds and cooling to 60°C for 1 minute.

### Cytokine/chemokine Beadarray

25 ul media (secretion) derived from human primary adipocytes were used for cytokine/chemokine (50-plex) analysis using the human cytokine/chemokine 27-plex and 23-plex multiplex assay kit (Bio-rad, The Netherlands). Multiplex (27-plex) IL-1ß, IL-1ra, IL-2, IL-4, IL-5, IL-6, IL-7, IL-8, IL-9, IL-10, IL-12 (p 70), IL-13, IL-15, IL-17, B-FGF, Eotaxin, G-CSF, GM-CSF, IFN-γ, IP-10, MCP-1 (MCAF), MIP-1α, MIP-1ß, PDGF-BB, RANTES, TNF-α, VEGF, and multiplex (23-plex) IL-1αIL-2RαIL∼3IL-12P40), IL-16, IL-18, CTACK, GRO-αHGF, ICAM-1, IFN-α2, LIF, MCP-3, M-CSF, MIF, MIG, β-NGF, SCF, SCGF-β, SDF-1α, TNF-β, TRAIL and VCAM-1 were performed in Luminex according to the manufacturer's instructions.

### Transmission Electron Microscopy (TEM)

To make slides from Preadipocytes, three days after differentiation and adipocytes, preadipocytes were cultured in six wells dishes. Each two wells were meant for one specific phase. Media were discarded and cells were washed three times with PBS. The cells were fixed for 24 h with 2% Paraformaldehyde and 1% glutaraldehyde at 4°C. Fixation solutions was removed and the cells were then washed with PBS and 6.8% sucrose (pH 7.4). Post fixation was done at 4°C in 1% osmiumtetroxide (OsO4) dissolved in 0.1 M PBS containing 1.5% potassiumhexacyanoferrate (II)trihydrate (Merck, Darmstadt, Germany). Then the slides were washed with distilled water, dehydrated through a graded series of ethanol and finally embedded in EPON 812 (Serva Feinbiochemica, Heidelberg Germany). Ultrathin sections were cut on a Sorvvall microtome (Sorvall, Newton, Con, USA) and concentrated with uranyl acetate and lead citrate and ultimately analyzed in a Philips CM 100/80 kv TEM (Philips, Eindhoven, The Netherlands).

### Confocal Microscopy/Immunoelectron Microscopy

Human primary adipocytes cultured in six wells and each well was incubated with Antibodies against: C-reactive protein (CRP; mAb CRP-8, Sigma, USA), and Perilipin (PLIN; polyclonal antibody, mAb, Abcam, USA). Bound antibodies were detected with an Alexa 647-coupled goat anti-mouse antibody or swine anti-rabbit in case of perilipin (Dako, Glostrup, Denmark). Lipids and Nucleus were stained with FITC and DAPI respectively (ValaScience). As control, adipocytes slides were not incubated with the primary antibodies, but with IgG isotype 1 and the rest of procedure remained unchanged. Specific antibody binding was visualized using a high resolution Leica SP2 AOBS Confocal laser scan microscope (CLSM) (Leica Microsystems Nederland BV, The Netherlands), and images were obtained with Leica confocal software.

The same approach was used for immunoelectron microscopy except Bound antibodies were detected with coupled goat anti-mouse 15 nm gold particles.

### Cell Migration Assay

Human primary preadipocytes were cultured in 12-well plate and differentiated into adipocytes as mentioned in the paragraph of cell culture (Materials and Methods). Cell Migration Assay was performed in a Boyden chamber with a PET membrane (5 µm pore size) inserts (Millipore) in a 12-well plate. The insert was hanged on each well with attached adipocytes of 12-well plate. Blood was collected from normal healthy volunteers after written informed consent, by venipuncture into 6×10 ml Lithium Heparin tubes (in each experiment, Blood from one healthy individual was used). Subsequently, lymphocytes were isolated from blood using lymphoprep according to the manual (Axis-Shield).

CD4 T cells were stained with FITC labeled CD4 (IQ products) for 30 min. and after washing steps, lymphocytes subjected to the MoFLo cell sorting to purify CD4+ T cells.

CD4+ T cells were typically reached a purity of 95–99% using flow cytometry. This CD4+ cell suspension was used in *migration* experiments. CD4+ cell (2.8×10^5^) suspension is placed in upper chamber of insert. The cells (in the both sides of insert) were cultured in the absence of serum and incubated at 37°C for 10 h. The migrated cells were collected and the number of migrated cells was obtained by cell counting using Burker-Turk chamber. One migration experiment was performed with the plated CD4+ cells (7×10^5^) suspension on top of a culture insert in the absence of serum and incubated at 37°C for 30 h. Monoclonal antibodies against human MCP-1 and IP-10 (R & D system) were used to neutralize the effect of these two chemoattractants in migration assay. Adipocytes were treated with LPS (200 ng/ml) in both migration and co-stimulation experiments. As control in experiments, we used 1- only media, 2- media and LPS and 3- media and IgG isotype 1. The study had the approval of the local Medical ethical committee (University Medical Center Groningen).
